# Ethanol Extract of *Atractylodes macrocephala* Protects Bone Loss by Inhibiting Osteoclast Differentiation

**DOI:** 10.3390/molecules18077376

**Published:** 2013-06-24

**Authors:** Hyunil Ha, Hyosun An, Ki-Shuk Shim, Taesoo Kim, Kwang Jin Lee, Youn-Hwan Hwang, Jin Yeul Ma

**Affiliations:** KM-Based Herbal Drug Research Group, Korea Institute of Oriental Medicine, Daejeon 305-811, Korea

**Keywords:** *Atractylodes macrocephala*, osteoporosis, RANKL, osteoclast

## Abstract

The rhizome of *Atractylodes macrocephala* has been used mainly in Traditional Chinese Medicine for invigorating the functions of the stomach and spleen. In the present study, we investigated the inhibitory effect of the 70% ethanol extract of the rhizome of *Atractylodes macrocephala* (AMEE) on osteoclast differentiation. We found that AMEE inhibits osteoclast differentiation from its precursors induced by receptor activator of nuclear factor-κB ligand (RANKL), an essential cytokine required for osteoclast differentiation. AMEE attenuated RANKL-induced activation of NF-κB signaling pathway, subsequently inhibiting the induction of osteoclastogenic transcription factors, c-Fos and nuclear factor of activated T cells cytoplasmic 1. Consistent with the *in vitro* results, administration of AMEE protected RANKL-induced bone loss in mice. We also identified atractylenolide I and II as active constituents contributing to the anti-osteoclastogenic effect of AMEE. Taken together, our results demonstrate that AMEE has a protective effect on bone loss via inhibiting osteoclast differentiation and suggest that AMEE may be useful in preventing and treating various bone diseases associated with excessive bone resorption.

## 1. Introduction

Osteoporosis, a metabolic disease characterized by low bone mass and microarchitectural deterioration of bone tissue, is a major health problem of aging population, especially in postmenopausal women [1]. Bone is a dynamic organ that undergoes continuous remodeling through the cooperative action of osteoclasts that resorb old bone and osteoblasts that form new bone. This remodeling process is necessary to repair damaged bone and to maintain mineral homeostasis. However, an imbalance of bone resorption over bone formation causes bone loss in pathological conditions such as osteoporosis and rheumatoid arthritis [2]. 

Osteoclasts are multinucleated giant bone-resorbing cells differentiated from monocyte/macrophage lineage precursor cells. Receptor activator of NF-κB ligand (RANKL) is an essential cytokine that promotes the differentiation of osteoclast precursor cells into osteoclasts [3,4]. RANKL binding to its receptor RANK on the cell surface of osteoclast precursors induces the recruitment of adaptor molecules such as tumor necrosis factor receptor-associated factor 6, which stimulates the activation of mitogen activated protein (MAP) kinases and transcription factors including NF-κB, c-Fos, and nuclear factor of activated T cells cytoplasmic 1 (NFATc1) [5,6]. These transcription factors play a crucial role in osteoclast differentiation [7,8,9]. It was also reported that sequential activation of NF-κB, c-Fos, and NFATc1 is required for proper osteoclast differentiation [6]. 

Although anti-resorptive agents such as bisphosphonates and the bone anabolic agent parathyroid hormone have been established to treat osteoporosis, the currently available pharmaceutical products have various disadvantages that limit their efficacy. Recently, there is an increasing interest in natural products including herbal extracts and food supplements for the prevention and treatment of osteoporosis [1,10]. In the screening of herbal extracts for anti-osteoclastogenic activity, we found that the 70% ethanol extract of the rhizome of *Atractylodes macrocephala* (AMEE) has an inhibitory effect on osteoclast differentiation. 

The rhizome of *A. macrocephala* has been widely used in traditional Chinese medicine for invigorating the functions of the stomach and spleen, benefiting vital energy, and eliminating dampness [11]. It has been reported that extracts of the rhizome of *A. macrocephala* possess various pharmacological properties including anti-obesity [12], anti-inflammatory [13], antioxidant [14], neuroprotective [15], immunostimulant [16], and anti-allergic [17] effects. However, its bone protective effect has not been studied. In the present study, the inhibitory effect and underlying mechanism of AMEE on osteoclast differentiation were investigated. In addition, its bone protective effect was evaluated in a murine model of bone loss by RANKL injection.

## 2. Results and Discussion

### 2.1. AMEE Inhibits Osteoclast Differentiation in Bone Marrow Cell-Osteoblast Coculture

**Figure 1:**
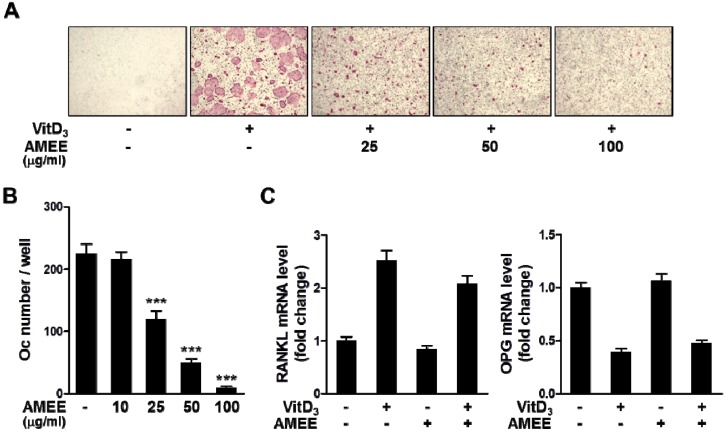
Effect of AMEE on osteoclast differentiation in bone marrow-osteoblast coculture. Mouse bone marrow cells and primary osteoblasts were cocultured with vehicle (dimethylsulfoxide, DMSO) or AMEE in the presence or absence of VitD_3_ (10 nM) for 6 days. (**A**) Cells were fixed and stained for tartrate-resistant acid phosphatase (TRAP). (**B**) TRAP-positive multinucleated cells containing more than three nuclei were counted as osteoclasts. *** *p* < 0.001 versus vehicle-treated control. (**C**) Osteoblasts were pretreated with AMEE (100 μg/mL) for 3 h and stimulated with VitD_3_ (10 nM) for 24 h. RANKL and OPG mRNA levels were analyzed by real-time quantitative polymerase chain reaction (qPCR).

We investigated whether AMEE inhibits osteoclast differentiation in bone marrow cell-osteoblast coculture system, in which calciotropic factors such as 1α,25-dihydroxyvitaminD3 (VitD_3_) and IL-1 promote osteoclast differentiation from bone marrow cells by increasing RANKL expression and/or decreasing its decoy receptor osteoprotegerin (OPG) expression in osteoblasts [2]. Treatment of the cocultures with VitD_3_ for 6 days induced osteoclast differentiation, which was inhibited by AMEE in a dose-dependent manner (Figure 1A,B). We next investigated whether AMEE affects the expression of RANKL and OPG in osteoblasts. VitD_3_ increased RANKL mRNA levels and decreased OPG mRNA levels. AMEE did not affect RANKL and OPG mRNA expression in basal and VitD_3_-stimulated osteoblasts at a concentration of 100 μg/mL that markedly inhibited osteoclast differentiation (Figure 1C). 

### 2.2. AMEE Inhibits RANKL-Induced Osteoclastogenesis from Osteoclast Precursors

**Figure 2:**
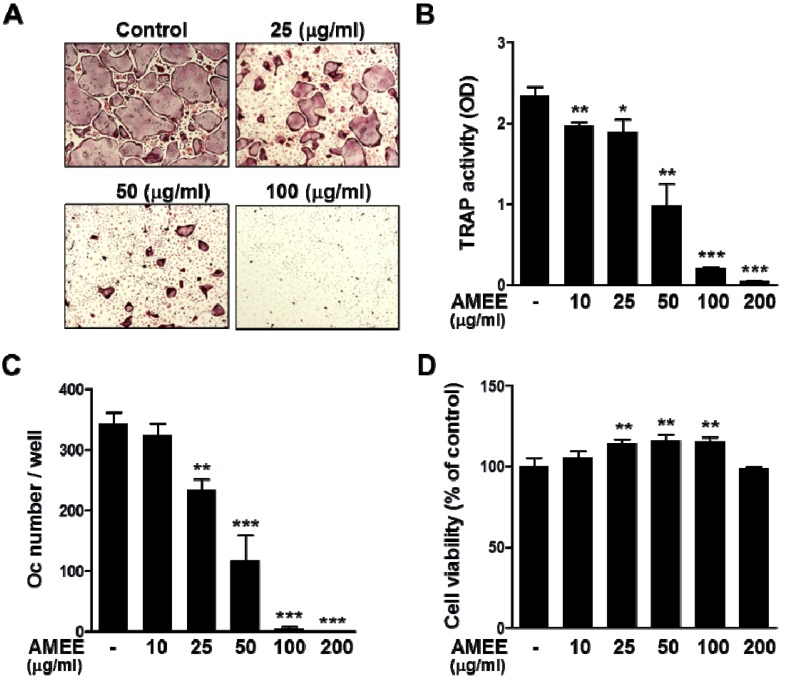
Effect of AMEE on RANKL-induced osteoclast differentiation in BMMs. BMMs were cultured with vehicle or AMEE in the presence of M-CSF (60 ng/mL) and RANKL (100 μg/mL) for 4 days. (**A**) TRAP staining. (**B**) Total cellular TRAP activity. (**C**) Osteoclast number. (**D**) Effect of AMEE on the viability of BMMs was determined by Cell Counting Kit-8 (CCK-8) assay. * *p* < 0.05; ** *p* < 0.01; *** *p* < 0.001 *versus* vehicle-treated control.

Based on the results of coculture experiments, the possibility that AMEE might inhibit osteoclast differentiation primarily through direct actions on osteoclast precursor cells was examined. In the presence of M-CSF, RANKL is able to induce the differentiation of precursor cells, such as mouse bone marrow-derived macrophages (BMMs), into mature osteoclasts [3]. Consistent with the results of coculture experiments, AMEE dose-dependently inhibited RANKL-induced TRAP activity and osteoclast differentiation in BMM cultures (Figure 2A–C). AMEE did not exhibit any detrimental effect on the viability of BMMs at concentrations of 10–200 μg/mL but rather increased cell proliferation of BMMs at 25–100 μg/mL (Figure 2D).

### 2.3. AMEE Inhibits RANKL-Induced c-Fos and NFATc1 Expression in Osteoclast Precursors

**Figure 3:**
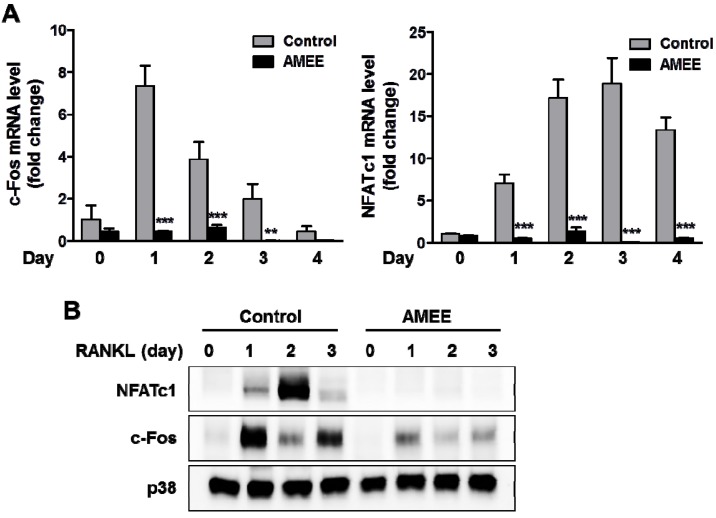
Effect of AMEE on RANKL-induced c-Fos and NFATc1 expression in BMMs. BMMs were cultured with vehicle or AMEE (100 μg/mL) in the presence of M-CSF (60 ng/mL) and RANKL (100 μg/mL) for the indicated days. (**A**) mRNA levels of c-Fos and NFATc1 were analyzed by qPCR. ** *p* < 0.01; *** *p* < 0.001 versus vehicle-treated control. (**B**) Protein expression levels of c-Fos and NFATc1 were determined by Western blot analysis. Total p38 was used as a loading control.

RANKL strongly induces NFATc1expression in osteoclast precursors, and NFATc1-deficient cells fail to differentiate osteoclasts in response to RANKL. Furthermore, ectopic overexpression of NFATc1 can induce the differentiation of osteoclast precursors into mature osteoclasts even in the absence of RANKL [9]. Thus, NFATc1 is thought to be the master transcription factor for osteoclast differentiation. The transcription factor c-Fos functions as a key upstream activator of NFATc1 during osteoclast differentiation through binding to the promoter region of NFATc1 [18]. To elucidate the molecular mechanism underlying the anti-osteoclastogenic effect of AMEE, we examined the effect of AMEE on the expression of c-Fos and NFATc1 during RANKL-induced osteoclast differentiation. RANKL increased c-Fos mRNA and protein expression reaching a peak at day 1, followed by the induction of NFATc1. Pretreatment with AMEE abrogated RANKL-induced c-Fos and NFATc1 induction, suggesting that the c-Fos pathway is a target of the anti-osteoclastogenic effect of AMEE (Figure 3A,B).

### 2.4. AMEE Attenuates RANKL-Induced NF-κB Activation in Osteoclast Precursors

**Figure 4:**
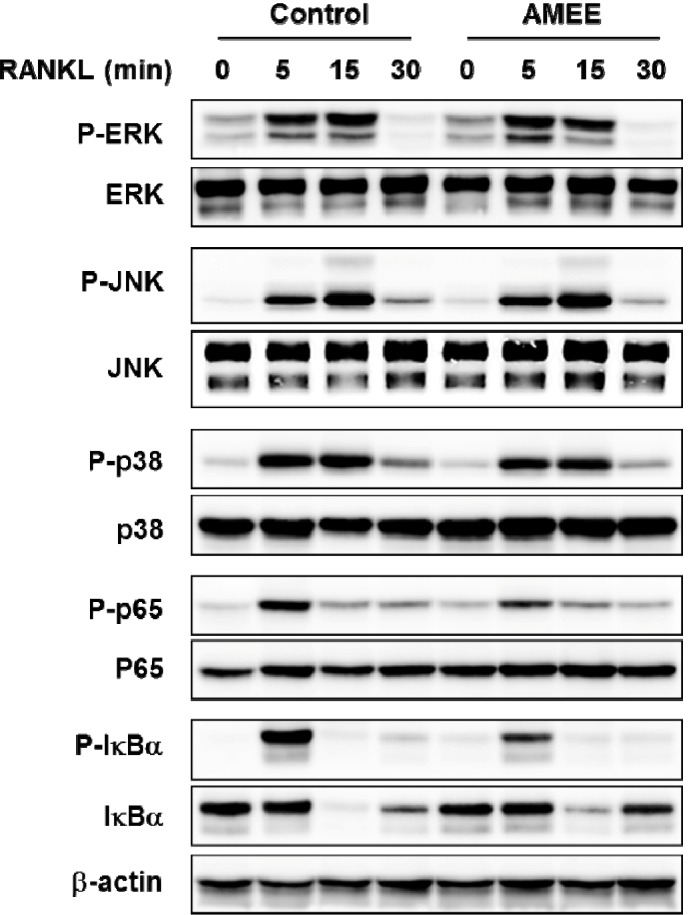
Effect of AMEE on the activation of MAP kinases and NF-κB in BMMs. BMMs were pretreated with AMEE (100 μg/mL) for 3 h and then stimulated with RANKL (100 ng/mL) for the indicated times. The total and phosphorylation levels of the indicated proteins were determined by Western blot analysis. β-actin was used as a loading control.

RANKL activates NF-κB and ERK, JNK, and p38 MAP kinases in osteoclast precursors, and these signaling pathways are implicated in c-Fos and NFATc1 induction as well as osteoclast differentiation [6,19,20,21]. Thus, we investigated whether AMEE could affect these signaling pathways. Stimulation of BMMs with RANKL transiently increased the phosphorylation of ERK, JNK, and p38 MAP kinases reaching a peak at 15 min. Pretreatment with AMEE did not affect the phosphorylation of these MAP kinases (Figure 4). The classical NF-κB signaling pathway depends on IκB kinase complex-mediated IκBα phosphorylation and degradation, allowing free NF-κB heterodimer containing the p50 and p65 subunits to translocate to the nucleus and activate transcription of target genes containing κB regulatory elements. In addition to IκBα degradation, various post-translational p65 modifications such as phosphorylation and acetylation can regulate NF-κB transcriptional activity [22]. Previous studies have shown that p65 phosphorylation on Ser536 in the transactivation domain promotes its transcriptional activity [23,24]. In the present study, RANKL stimulated p65 phosphorylation on Ser536 as well as IκBα phosphorylation and degradation, which was attenuated by AMEE (Figure 4). Therefore, it is likely that AMEE inhibits RANKL-induced c-Fos expression by suppressing NF-κB activation, thereby inhibiting osteoclastogenesis. 

### 2.5. AMEE Attenuates RANKL-Induced Bone Loss in Mice

**Figure 5:**
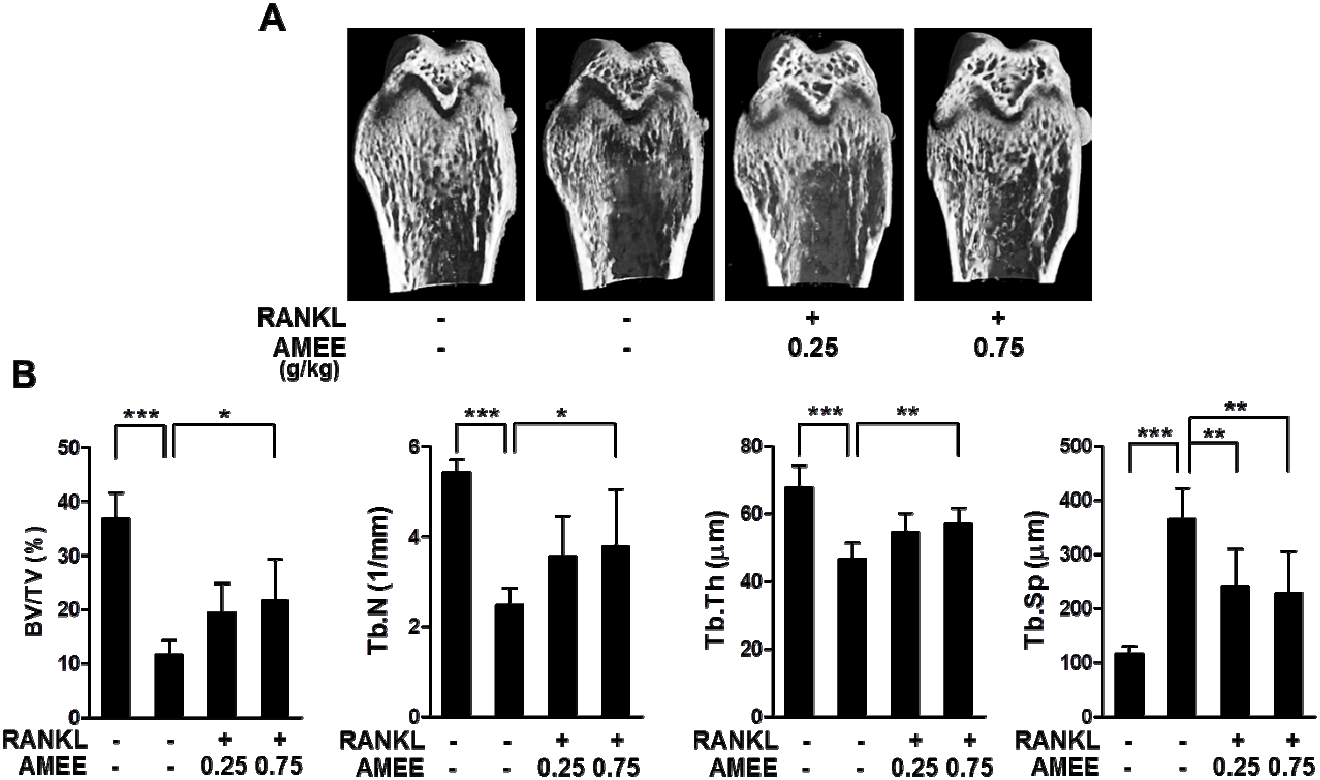
Effect of AMEE on RANKL-induced bone loss *in vivo*. Mice were orally administrated with AMEE (0.25 and 0.75 g/kg) at 12 h intervals for 5 consecutive days (day -2 to 2), and RANKL (1 mg/kg) was injected intraperitoneally on days 0 and 1. The femora were collected on day 3 and analyzed by micro-CT scanning. (**A**) Representative micro-CT images of the distal femora. (**B**) Trabecular bone volume/tissue volume (BV/TV), trabecular number (Tb. N), trabecular thickness (Tb. Th), and trabecular separation (Tb. Sp) at the distal femoral metaphysis. * *p* < 0.05; ** *p* < 0.01; *** *p* < 0.001.

We next investigated whether AMEE has a protective effect on bone loss associated with enhanced osteoclastic bone resorption. Mice were orally pretreated with AMEE and then injected intraperitoneally with RANKL. The RANKL injection led to a severe trabecular bone loss at the distal femoral metaphysis (Figure 5A). Micro-computed tomography (micro-CT) analysis showed a profound decrease in trabecular bone volume, thickness, and number, with a concomitant decrease in trabecular separation in the RANKL-injected mice (Figure 5B). Oral administration of AMEE (0.75 g/kg) significantly attenuated the changes in trabecular architecture induced by RANKL injection (Figure 5A,B). It has been reported that the intraperitoneal administration of RANKL rapidly induces trabecular bone loss through stimulating osteoclast differentiation and function, without affecting osteoblastic bone formation [25]. Thus, these observations suggest that AMEE can protect bone loss by suppressing osteoclastic bone resorption. 

### 2.6. Atractylenolide I Inhibits RANKL-Induced Osteoclast Differentiation

**Figure 6:**
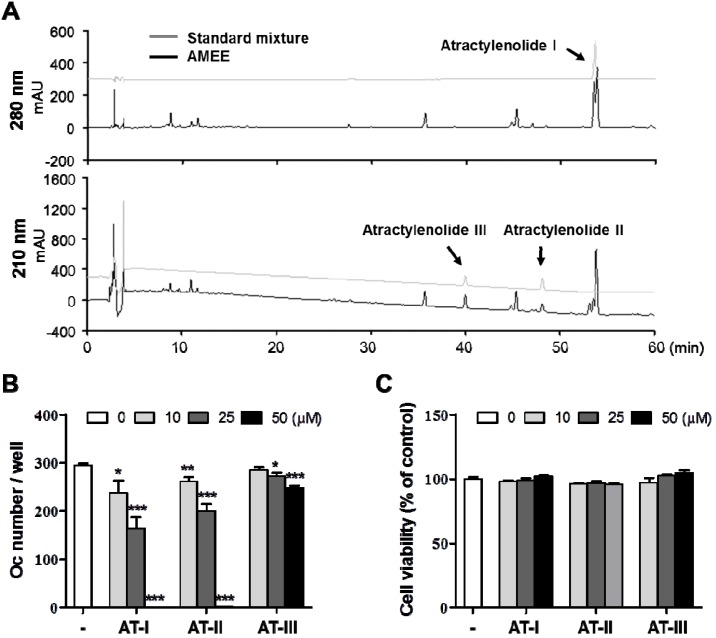
Phytochemical profile of AMEE and the anti-osteoclastogenic effects of atractylenolides. (**A**) HPLC chromatograms of a standard mixture of atractylenolide I, II, and III and AMEE at 210 and 280 nm. (**B**) BMMs were cultured with vehicle (DMSO) or atractylenolide I (AT-I), II (AT-II), and III (AT-III) in the presence of M-CSF (60 ng/mL) and RANKL (100 μg/mL) for 4 days. TRAP-positive multinucleated cells containing more than three nuclei were counted as osteoclasts. * *p* < 0.05; ** *p* < 0.01; *** *p* < 0.001 versus vehicle-treated control. (**C**) Effects of atractylenolide I, II, and III on the viability of BMMs were determined by CCK-8 assay.

Since AMEE exhibited a potent anti-osteoclastogenic action *in vitro* and *in vivo*, it is worth identifying the active constituents responsible for the anti-osteoclastogenic action. Eudesmane-type sesquiterpenoids such as atractylenolide I, II, and III have been reported to be major components of the rhizome of *A. macrocephala* [26,27,28]. In accordance with this, atractylenolide I, II, and III were identified from AMEE by high performance liquid chromatography class=st1> (HPLC) analysis, based on their HPLC retention times and UV absorption spectra in comparison with those of corresponding authentic standards (Figure 6A). These sesquiterpenoids possess multiple pharmacological properties including anti-inflammatory [29], anti-allergic [30], anti-tumoral [31], anti-hepatotoxicity [32], and gastroprotective [33] activities. We next examined whether these sesquiterpenoids affect osteoclast differentiation. Atractylenolide I showed the strongest inhibitory effect on RANKL-induced osteoclast differentiation in BMM cultures at concentrations without affecting the cell viability. The inhibitory effect of atractylenolide II was similar to that of atractylenolide I, whereas atractylenolide III showed much less inhibitory potency compared with atractylenolide I and II (Figure 6B,C). These observations suggest that atractylenolide I and II might be active constituents contributing to the anti-osteoclastogenic effect of AMEE. Atractylenolide III contains a hydroxyl group at the 8-position, while atractylenolide I and II lack a hydroxyl group at the 8-position. Thus, the hydroxyl group at the 8-position is most likely to contribute to the differential inhibitory effects of these sesquiterpenoids. The molecular mechanisms underlying the differential effects of atractylenolides remain to be elucidated.

## 3. Experimental

### 3.1. Reagents and Antibodies

α-modified minimal essential medium (α-MEM), fetal bovine serum (FBS), and antibiotics (100 U/mL penicillin and 100 μg/mL streptomycin) were purchased from Thermo Fisher Scientific Inc. (Rockford, IL, USA). VitD_3_ was purchased from Sigma-Aldrich (St. Louis, MO, USA). Atractylenolide I, Atractylenolide II, and atractylenolide III were obtained from ChemFaces (Wuhan, China). M-CSF was kindly provided by Dr. Yongwon Choi (University of Pennsylvania School of Medicine). Recombinant soluble RANKL was prepared as described in a previous report [25]. Antibodies against phospho-ERK1/2 (Thr202/Tyr204), ERK, phospho-JNK1/2 (Thr183/Tyr185), JNK, phospho-p38 (Thr180/Tyr182), p38, phospho-IκBα (Ser32), IκBα, phospho-p65 (Ser536), and p65 were purchased from Cell singling Technology (Danvers, MA, USA). Antibodies against NFATc1 and c-Fos were obtained from Santa Cruz biotechnology (Santa Cruz, CA, USA). Antibody against β-actin was from Sigma-Aldrich. 

### 3.2. Preparation of AMEE

Air-dried rhizome of *A. macrocephala* was purchased Yeongcheon Oriental Herbal Market (Yeongcheon, Korea). A voucher specimen (No. E59) was deposited in the herbal bank of KM-Based Herbal Drug Research Group, Korea Institute of Oriental Medicine. The rhizome of *A. macrocephala* (30 g) was ground to a fine powder and extracted by maceration with 300 mL of 70% ethanol at 40 °C for 24 h in a shaking incubator. The extract was filtrated through a testing sieve (150 μm), evaporated on a rotatory evaporator, and finally dried by a freeze dryer to prepare AMEE. For *in vitro* experiments, AMEE was resolved in DMSO and centrifuged at 10,000 × *g* for 5 min. 

### 3.3. Cell Culture

BMMs from male ICR mice were prepared as described previously [34] and cultured in α-MEM complete medium containing 10% FBS and antibiotics in the presence of M-CSF (60 ng/mL). To induce osteoclast differentiation from BMMs, BMMs (1 × 10^4^ cells/well) were cultured with M-CSF (60 ng/mL) and RANKL (100 ng/mL) for 4 days in a 96-well plate. The viability of BMMs was determined 2 days after culturing BMMs with M-CSF and AMEE by using CCK-8 assay (Dojindo Molecular Technologies Inc., Rockville, MD, USA). Mouse osteoblasts were obtained from calvariae of newborn ICR mice by enzymatic digestion as described previously [34] and cultured in α-MEM complete medium. For osteoclast differentiation assay, mouse bone marrow cells (3 × 10^5^ cells/well) and osteoblasts (2 × 10^4^ cells/well) were cocultured for 6 days with VitD_3_ (10 nM) in a 48-well culture plate. All cultures were replenished with fresh medium containing the same supplements on day 3. 

### 3.4. TRAP Activity and Staining

Cells were fixed in 10% neutral buffered formalin for 10 min at room temperature and then permeabilized with 0.1% Triton X-100 in PBS for 5 min. To measure total cellular TRAP activity, permeabilized cells were incubated with 200 μL of TRAP buffer (50 mM sodium tartrate and 0.12 M sodium acetate, pH 5.2) containing 1 mg/mL of *p*-nitrophenyl phosphate at 37°C for 5 min. The reaction mixtures were transferred into new plates containing an equal volume of 0.1 N NaOH, and the optical density was determined at 405 nm. For TRAP staining, cells were stained with TRAP buffer containing naphthol AS-MX phosphate (0.1 mg/mL) and fast red violet LB salt (0.5 mg/mL). After staining, cells were washed with distilled water and observed under a light microscope (100× magnification). 

### 3.5. QPCR Analysis

Total RNA was isolated with RNA-spin total RNA Extraction Kit (Bioneer, Daejeon, Korea), and cDNA was synthesized from 1 μg of total RNA using AccuPower RT-PreMix (Bioneer). SYBR green-based qPCR amplification was performed on the ABI 7500 Real-Time PCR System (Applied Biosystems, Foster City, CA, USA) using AccuPower GreenStar qPCR Master Mix (Bioneer). The primer sequences were previously described [34]. All reactions were run in triplicate, and target gene expression was determined according to the 2-ΔΔCT method using Hprt as a reference gene.

### 3.6. Western Blot Analysis

Cells were washed twice with ice-cold PBS and lysed in RIPA buffer (Millipore, Temecula, CA, USA) with protease and phosphatase inhibitor cocktails (Roche Applied Science, Indianapolis, IL, USA) at 4°C. The cell lysates were centrifuged at 10,000 × *g* for 10 min at 4 °C, and the supernatants were collected. Protein concentration of cell lysates was determined using BCA Protein Assay Kit (Thermo Fisher Scientific Inc.). Protein samples (30 μg) were resolved by 10% sodium dodecyl sulfate-polyacrylamide gel electrophoresis and transferred to a polyvinylidene fluoride membrane (Millipore). The membrane was blocked with 5% non-fat dry milk in TBST (10 mM Tris-HCl [pH 7.5], 150 mM NaCl, and 0.1% Tween 20) for 1 h at room temperature and probed overnight with appropriate primary antibodies (1/1000 dilution) at 4 °C. After washing with TBST three times for 10 min each, the membranes was incubated with horseradish peroxidase-conjugated secondary antibodies (1/5000 dilution; Thermo Fisher Scientific Inc.) for 1 h at room temperature and washed with TBST three times for 10 min each. Chemiluminescent signals were detected using a Luminescent Image Analyzer LAS-4000 (Fuji Photo Film Co., Tokyo, Japan) with SuperSignal West Femto Maximum Sensitivity Substrate (Thermo Fisher Scientific Inc.).

### 3.7. Animal Experiments and Micro-CT Analysis

Animal experiments were performed according to the Guide for the Care and Use of Laboratory Animals of the National Institutes of Health and approved by the Institutional Animal Care and Use Committee at Korea Institute of Oriental Medicine. 6-week-old male ICR mice (Orient Bio Inc., Seoul, Korea) were housed under constant environmental conditions (22 ± 1 °C, 55 ± 10% relative humidity, and 12 h/12 h light/dark cycle) with free access to food and water. After acclimatization for 1 week, mice (n = 6 per group) were orally administered with vehicle (distilled water) or AMEE (0.25 and 0.75 g/kg of body weight) twice daily for 5 consecutive days beginning on day -2. RANKL (1 mg/kg of body weight) or PBS was injected intraperitoneally on days 0 and 1. The right femora were collected on day 3 and fixed in 10% neutral buffered formalin. Micro-CT scanning was performed with the SMX-90CT system (Shimadzu, Kyoto, Japan). Scans then were integrated into 3D voxel images and reconstructed by the VG Studio MAX 1.2.1 program (Volume Graphics, Heidelberg, Germany). The regenerated trabecular bone volume/tissue volume, number, thickness, and separation were calculated with TRI/3D-BON (RATOC System Engineering, Kyoto, Japan).

### 3.8. HPLC Analysis

HPLC analysis was performed using a Dionex Ultimate 3000 HPLC system (Thermo Fisher Scientific Inc.) The output signal of the detector was recorded using a Chromeleon data acquisition system (Dionex version 7.0.1.272). The chromatographic separation was carried out using RS-tech C_18_ column (4.6 mm × 250 mm, 5 μm), and column temperature was kept at 40 °C. The composition of the mobile phases were 0.1% trifluoroacetic acid in deionized water (A) and acetonitrile (B) with gradient elution as follows: 0–50 min, 10%–70% B; 50–60 min, 70%–70% B. Flow rate and injection volume were 1.0 mL/min and 10 μL, respectively. The chromatograms were obtained at 210 and 280 nm. Standard compounds (Atractylenolide I, Atractylenolide II, and Atractylenolide III; each 200 μg/mL) and AMEE (50 mg/mL) were dissolved in methanol and filtered through a 0.2 μm syringe filter prior to injection for HPLC analysis.

### 3.9. Statistical Analysis

Statistical difference was determined by Student’s *t* test for two-group comparisons and by one-way analysis of variance followed by Dunnett’s test for multiple-group comparisons. A *p*-value less than 0.05 was considered statistically significant. All data are expressed as mean ± SD. Results except Figure 5B are representative of three experiments.

## 4. Conclusions

In summary, AMEE inhibited osteoclast differentiation by suppressing RANKL-induced sequential activation of NF-κB, c-Fos, and NFATc1 transcription factors in osteoclast precursors. In addition, AMEE protected RANKL-induced bone loss *in vivo*. Furthermore, we also found that atractylenolide I and II, major constitutes of AMEE, inhibit RANKL-induced osteoclast differentiation. Collectively, this study has demonstrated the inhibitory effect and action mechanism of AMEE on bone loss. Given the important role of RANKL in pathological bone destruction, our findings suggest that AMEE may be useful in preventing and treating various bone diseases associated with excessive bone loss. 
